# Risk factors for necrotizing enterocolitis in preterm infants: a 1:1 matched case-control study

**DOI:** 10.3389/fped.2026.1807022

**Published:** 2026-03-25

**Authors:** Lie Huang, Jianhui Wang, Xiaomei Fu, Meile Cheng, Xi Xu

**Affiliations:** 1 Department of Pediatrics, The First People’s Hospital of Neijiang, Neijiang, Sichuan, China; 2 Department of Neonatology, Children’s Hospital of Chongqing Medical University, Chongqing, China; 3 Department of Neonatology, The First People’s Hospital of Yinchuan, Yinchuan, China; 4 Department of Science and Education, The First People’s Hospital of Neijiang, Neijiang, Sichuan, China

**Keywords:** infants, maternal gestational diabetes, necrotizing enterocolitis, risk factors, ROC (receiver operating characteristic curve)

## Abstract

**Impact:**

This study demonstrates that neonatal sepsis, blood transfusion, combined antibiotic use, asphyxia, and maternal gestational diabetes (GDM) are independent risk factors for NEC. By utilizing a 1:1 matched case-control design, we effectively eliminated the confounding effects of gestational age and birth weight, revealing critical perinatal drivers of the disease. The resulting prediction model (AUC = 0.937) provides a robust tool for early screening. These findings underscore the urgent need for enhanced antibiotic stewardship and rigorous maternal GDM management to reduce NEC incidence and improve outcomes in high-risk preterm infants.

**Background:**

Identifying independent clinical risk factors for necrotizing enterocolitis (NEC) facilitates early screening of high-risk preterm infants and enables the development of targeted prevention strategies.This retrospective study explored the influencing factors of NEC and applied logistic regression analysis to identify the key contributors to NEC onset, aiming to provide evidence for clinical diagnosis and treatment.

**Methods:**

A 1:1 matched case-control study was conducted, involving 172 preterm infants with NEC and 172 controls matched by GA (±1 week), BW (±100 g), and admission time. Univariate and multivariable logistic regression analyses were performed to identify independent risk factors.

**Results:**

A total of 172 preterm infants with NEC (study group) and 172 matched non-NEC preterm infants (control group) were enrolled in this retrospective case-control study. Univariate analysis revealed significant differences between the two groups in preterm premature rupture of membranes (PPROM), gestational diabetes mellitus (GDM), combined antibiotic use, blood product transfusion, neonatal sepsis, and neonatal asphyxia (all *P* < 0.05). Multivariate logistic regression analysis identified independent risk factors for NEC in preterm infants, including neonatal sepsis (OR = 2.522, 95%CI: 1.403–4.538, *P* = 0.002), blood transfusion (OR = 2.18, 95%CI: 1.245–3.809, *P* = 0.008), neonatal asphyxia (OR = 1.887, 95%CI: 1.085–3.274, *P* = 0.024), GDM (OR = 1.824, 95%CI: 1.102–3.020, *P* = 0.018), and combined antibiotic use (OR = 1.976, 95%CI: 1.305–2.972, *P* = 0.001). The Hosmer-Lemeshow test confirmed good fit of the regression model (*χ*^2^ = 13.152, *P* = 0.071). Receiver operating characteristic (ROC) curve analysis showed that the risk prediction model constructed with these independent factors had an area under the curve (AUC) of 0.937 (95%CI: 0.915–0.960), with a sensitivity of 93.8% and specificity of 79.5%.

**Conclusion:**

Sepsis, blood transfusion, combined antibiotics, asphyxia, and maternal GDM are key independent predictors of NEC. Clinicians should implement targeted monitoring and stewardship, particularly regarding antibiotic use and GDM management, to mitigate NEC risk.

## 1 Introduction

Necrotizing enterocolitis (NEC) is a severe intestinal necrotic syndrome that affects the immature intestines of neonates—particularly preterm infants and low birth weight infants (LBWIs). Its incidence is approximately 1‰ (1), reaching 11% in very low birth weight infants (VLBWIs) (2) and as high as 22% in extremely low birth weight infants (ELBWIs) (3). Clinically, it presents with nonspecific manifestations such as abdominal distension or pain, hematochezia, vomiting, feeding intolerance, respiratory distress, lethargy, and instability in body temperature and vital signs (4). A hallmark of NEC is its acute onset and rapid progression, contributing to substantial fatality rates, particularly in preterm infants.Notably, while advances in neonatal intensive care have improved the survival rates of preterm and low-birth-weight infants, this progress has been paralleled by a concomitant increase in NEC incidence (5). Recent meta-analyses confirm the substantial clinical burden of NEC, with an overall incidence of 2%–7% among preterm infants and mortality rates ranging from 23.5% to 50.9% (6).

The pathophysiology of NEC remains incompletely elucidated, but it is widely recognized as a multifactorial disorder involving the synergistic interaction of multiple risk factors (7). Among these factors, both maternal and neonatal factors are potential risk factors for NEC development (8, 9). Studies have shown that early identification of NEC risk factors, targeted interventions, and timely diagnosis and treatment are crucial for reducing the incidence and mortality rates of NEC (10).

Traditionally, gestational age (GA) and birth weight (BW) have been identified as the most significant predictors of NEC (11), as lower GA and BW are inherently linked to greater physiological immaturity. However, the overwhelming influence of GA and BW often acts as a major confounding factor in clinical research, potentially masking the independent effects of other critical perinatal variables.To address these limitations, this study employed a 1:1 matched case-control design. By matching each NEC case with a control infant of nearly identical gestational age and birth weight, we aimed to eliminate these dominant confounders. Through this analysis, we seek to provide clearer clinical evidence for the early identification and targeted prevention of NEC in high-risk preterm populations.

## 2 Materials and methods

### 2.1 Population selection

This study adopted a 1:1 matched case-control design to investigate the independent risk factors for necrotizing enterocolitis (NEC) in preterm infants. We retrospectively reviewed the medical records of preterm infants admitted to the Department of Neonatology at the First People's Hospital of Yinchuan between January 2017 and December 2023.

Case Group: A total of 172 preterm infants (gestational age < 37 weeks) diagnosed with NEC (Bell's stage ≥ II) were included (12).

Control Group: To eliminate the dominant confounding effects of physiological maturity, each NEC case was matched with one non-NEC control infant based on the following matching criteria: (1) Gestational Age (GA): Difference ≤ ± 1 week. (2) Birth Weight (BW): Difference ≤100 g. (3) Admission Time: Admitted within the same 6-month period to control for variations in clinical practice.

Exclusion Criteria: Infants with congenital gastrointestinal malformations, hospital stays < 7 days, no enteral feeding before onset, death within 14 days of admission, or incomplete clinical records were excluded.The study protocol was approved by the Ethics Committee of the First People's Hospital of Yinchuan (Approval No. 2024102).

### 2.2 Variables

The collected data were categorized into four main types, as follows:

General clinical data of the preterm infants: sex,ethnicity,gestational age, whether small for gestational age (SGA), birth weight, 1-minute and 5-minute Apgar scores, feeding methods (breastfeeding), and mode of delivery (cesarean section).

Maternal-related factors: maternal age, meconium-stained amniotic fluid, preeclampsia, chorioamnionitis, fetal distress, premature rupture of membranes (PROM), multiple gestation, antenatal corticosteroid administration, and gestational comorbidities (including gestational diabetes mellitus, gestational hypertension, intrahepatic cholestasis of pregnancy, and maternal hypothyroidism).

Interventions before the onset of NEC: probiotic use before onset, antibiotic use duration, blood transfusion before onset, and umbilical vein catheterization.

Additionally, data on comorbidities were collected only if the diagnosis was established prior to the clinical or radiological onset of NEC. These comorbidities included neonatal respiratory distress syndrome (NRDS), sepsis, patent ductus arteriosus (PDA), neonatal asphyxia, cholestasis, and bronchopulmonary dysplasia (BPD).

Combined antibiotic use: Concurrent administration of two or more different classes of antibiotics within a single hospitalization day.

Feeding methods: Breastfeeding was defined as the consumption of the mother's own milk (MOM), provided either via direct breastfeeding or via bottle or nasogastric tube. Infants in the non-breastfeeding group were primarily fed with bovine-milk-based preterm formula.

### 2.3 Statistical analysis

All data were analyzed using SPSS 26.0 software. Quantitative data conforming to a normal distribution were presented as mean ± standard deviation (SD) and compared between groups using the independent samples t-test. Skewed distributed data were presented as median [interquartile range (IQR)] and analyzed using the Mann–Whitney U test. Categorical variables were described as frequency (n) and percentage (%), and compared using the chi-square test. Multivariate logistic regression analysis was performed to identify independent risk factors for NEC, with variables showing statistical significance in univariate analysis included as covariates. The predictive value was evaluated via receiver operating characteristic (ROC) curve analysis, and a risk prediction model was subsequently established. A *P*-value < 0.05 was considered statistically significant.

## 3 Results

**Figure 1:**
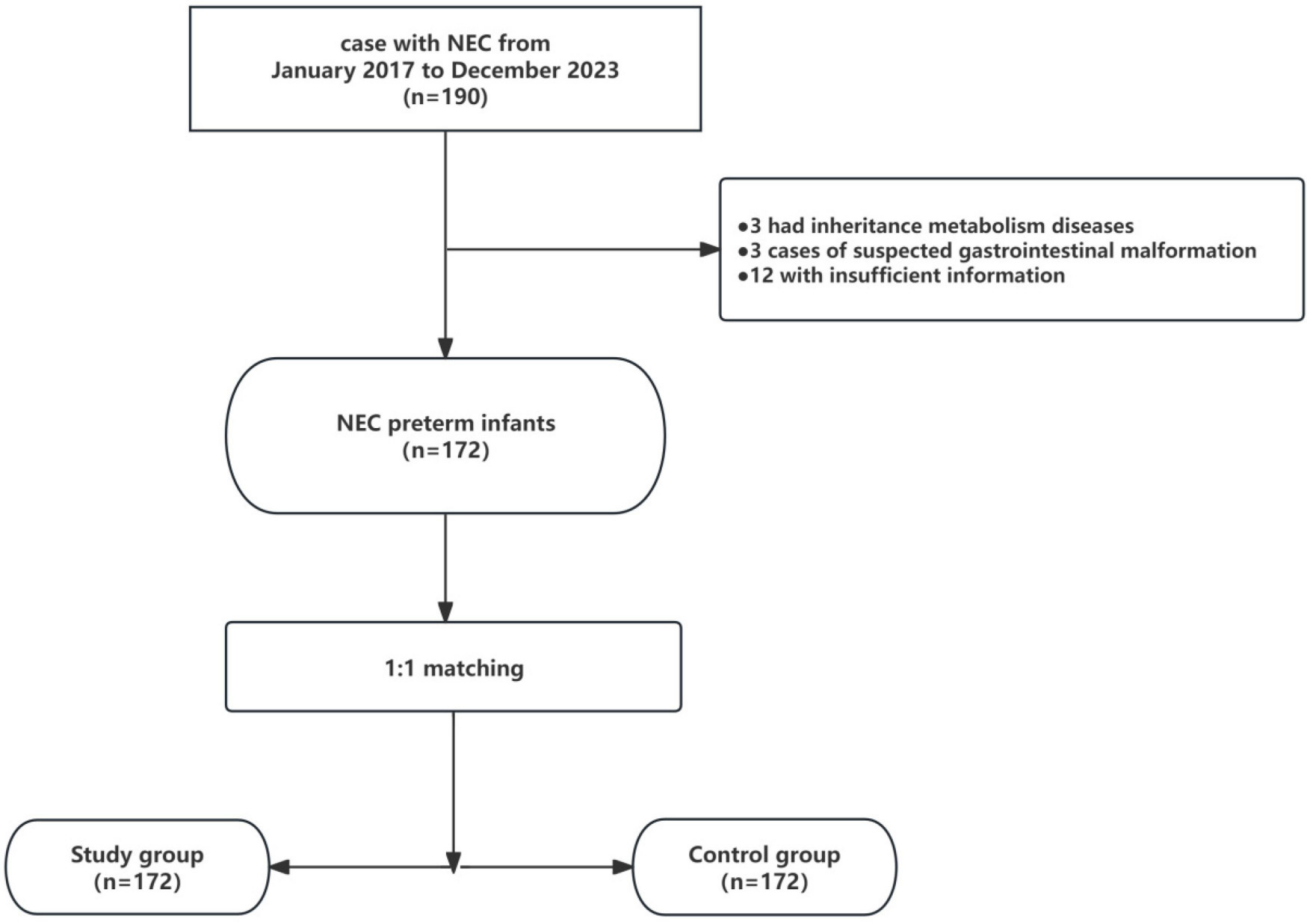
Flow chart of selection of the study population.

During the research period, a total of 172 preterm infants diagnosed with NEC were discovered. Figure 1 depicts the selection process of the NEC preterm infants included in this study.

### 3.1 Comparison of baseline clinical characteristics among preterm infants in the two groups

A total of 172 preterm infants with NEC (Study Group) and 172 matched non-NEC infants (Control Group) were enrolled. As shown in Table 1, through rigorous 1:1 matching, no statistically significant differences were observed between the two groups in terms of gestational age (GA) and birth weight (BW) (all *P* > 0.05; Table 1).

**Table 1:** Comparison of general data between the two groups .

General data	Study group (*n* = 172)	Control group (*n* = 172)	*P-value*	OR (95%CI)
GA (week), mean (SD)	35.0 ± 2.1	35.2 ± 1.8	0.645	-
Birth weight (g), mean (SD)	2,895 ± 836	2,910 ± 720	0.862	-
Gender (male), *n* (%)	99 (57.6)	106 (61.6)	0.448	0.844 (0.545–1.304)
Ethnicity (hui), *n* (%)	45 (26.1)	38 (22.1)	0.377	1.249 (0.751–2.037)
SGA, *n* (%)	19 (11)	21 (12.2)	0.736	0.893 (0.466–1.678)
Feeding methods (breastfeeding), *n* (%)	56 (32.5)	46 (26.7)	0.238	1.322 (0.823–2.087)
Delivery mode (cesarean), *n* (%)	100 (58.1%)	85 (49.4%)	0.105	1.422 (0.932–2.182)
Apgar. 1 min, IQR	9.0 [8.0∼10.0]	9.0 [8.0∼10.0]	0.248	-
Apgar. 5 min, IQR	10.0 [9.0∼10.0]	10.0 [9.0∼10.0]	0.675	-

GA, gestational age; SGA, small for gestational age; IQR, interquartile range.

### 3.2 Comparison of maternal-related factors between the two groups

With respect to maternal-related factors, statistically significant differences were identified between the study and control groups in preterm premature rupture of membranes (PROM) and gestational diabetes mellitus (GDM) (both *P* < 0.05). In contrast, no significant between-group differences were observed regarding maternal age, meconium-stained amniotic fluid, chorioamnionitis, antenatal steroid (ANS), fetal distress, preeclampsia, gestational comorbidities (e.g., gestational hypertension, intrahepatic cholestasis of pregnancy, maternal hypothyroidism), or multiple gestations (all *P* > 0.05; Table 2).

**Table 2:** Comparison of maternal-related factors between the two groups.

Maternal-related factors	Study group (*n* = 172)	Control group (*n* = 172)	*P-value*	OR (95%CI)
Maternal age (≥35 years), *n* (%)	30 (17.4)	19 (11)	0.09	1.71 (0.909–3.102)
Meconium-stained amniotic fluid, *n* (%)	28 (16.3)	32 (18.6)	0.57	0.851 (0.495–1.503)
Preeclampsia, *n* (%)	41 (23.8)	37 (21.5)	0.606	1.142 (0.693- 1.896)
Chorioamnionitis, *n* (%)	26 (15.1)	19 (11)	0.263	1.434 (0.774–2.635)
ANS, *n* (%)	116 (67.4)	123 (71.5)	0.412	0.825 (0.528–1.316)
Fetal distress, *n* (%)	23 (13.4)	30 (17.4)	0.296	0.731 (0.414–1.308)
PROM, *n* (%)	28 (16.3)	46 (26.7)	0.018	0.533 (0.312–0.902)
GDM, *n* (%)	40 (23.2)	20 (11.6)	0.004	2.303 (1.275- 4.129)
HDP, *n* (%)	26 (15.1)	20 (11.6)	0.342	1.353 (0.738–2.591)
ICP, *n* (%)	16 (9.3)	12 (7)	0.430	1.368 (0.6206–2.996)
Hypothyroidism, *n* (%)	8 (4.6)	9 (5.2)	0.803	0.883 (0.337–2.176)
Multiple gestation, *n* (%)	19 (11)	12 (7)	0.187	1.656 (0.807–3.499)

ANS, antenatal steroid; PROM, premature rupture of membranes; GDM, gestational diabetes mellitus; HDP, hypertensive disorders of pregnancy; ICP, intrahepatic cholestasis of pregnancy.

### 3.3 Comparison of pre-NEC clinical interventions between the two groups

An analysis of clinical interventions implemented prior to NEC onset across the two groups demonstrated statistically significant disparities in blood product transfusion (*P* < 0.05). In contrast, no meaningful between-group difference were detected regarding the utilization of umbilical venous catheterization, probiotic administration and duration of antibiotic use (Table 3).

**Table 3:** Comparison of prior interventions between the two groups.

Clinical interventions	Study Group (*n* = 172)	Control Group (*n* = 172)	*P-value*	OR (95%CI)
				
Probiotics, *n* (%)	10 (5.81)	17 (9.9)	0.161	0.562 (0.258–1.276)
Antibiotic use duration, IQR	10.00 [6.00, 15.00]	10.00 [6.00, 15.75]	0.957	-
blood product transfusion, *n* (%)	37 (21.5)	11 (6.4)	<0.001	4.011 (2.006−8.207)
UVC, *n* (%)	3 (1.7)	4 (2.3)	1^a^	1.341 (0.296–6.085)

IQR, interquartile range; UVC, umbilical venous catheterization.

^a^ Fisher's precision probability test.

### 3.4 Comparison of comorbidities between the two groups

Statistically significant differences were observed between the two groups in the incidences of neonatal sepsis and neonatal asphyxia (both *P* < 0.05). In contrast, no significant between-group differences were detected in the incidences of cholestasis, neonatal respiratory distress syndrome (NRDS), bronchopulmonary dysplasia (BPD), or patent ductus arteriosus (PDA) (all *P* > 0.05; Table 4).

**Table 4:** Comparison of comorbidities between the two groups.

Comorbidities	Study Group (*n* = 172)	Control Group (*n* = 172)	*P-value*	OR (95%CI)
NRDS, *n* (%)	7 (4.1)	3 (1.7)	0.335	0.418 (0.106–1.646)
Sepsis, *n* (%)	39 (22.7)	6 (3.5)	<0.001	8.113 (3.391–18.42)
PDA, *n* (%)	12 (7)	16 (9.3)	0.430	1.368 (0.621–2.996)
Neonatal asphyxia, *n* (%)	31 (18)	12 (7)	0.003	2.931 (1.456–5.727)
Cholestasis, *n* (%)	6 (3.5)	4 (2.3)	0.521*	1.518 (0.401–4.834)
BPD, *n* (%)	4 (3.5)	3 (2.3)	1*	0.745 (0.186–2.815)

NRDS, neonatal respiratory distress syndrome; PDA, patent ductus arteriosus; BPD, bronchopulmonary dysplasia. *: Fisher's precision probability test.

### 3.5 Multivariate analysis

**Figure 2:**
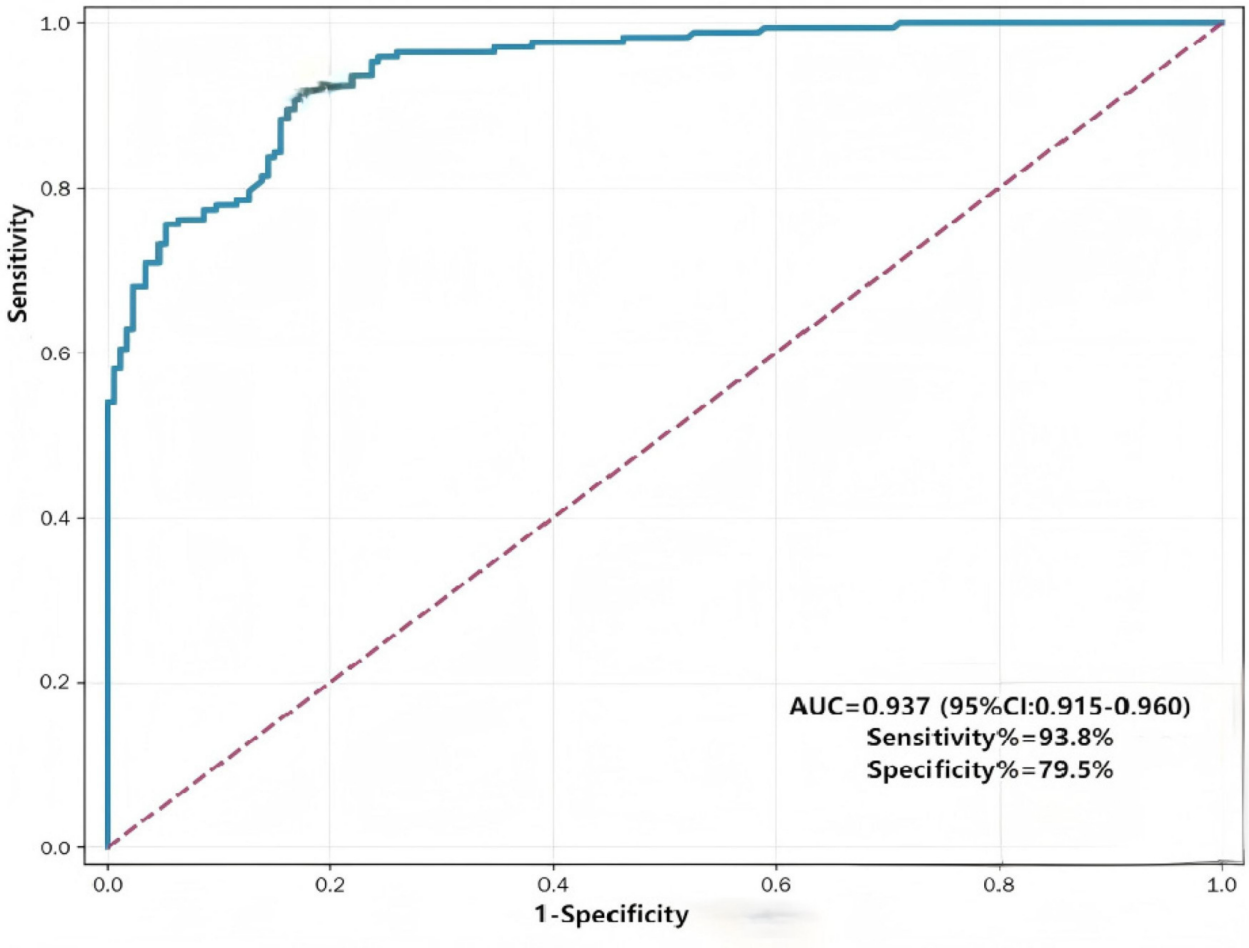
ROC curve for predicting influence factors of necrotizing enterocolitis. AUC = 0.937,sensitivity (93.8%) and specificity (79.5%).

Variables showing statistical significance in the univariate analysis were incorporated into a multivariable logistic regression model. After controlling for the interference of developmental maturity through the matched design, maternal gestational diabetes mellitus (GDM; OR = 1.824, 95% CI: 1.102–3.020) and combined antibiotic use (OR = 1.976, 95% CI: 1.305–2.972) were identified as independent risk factors for NEC. Additionally, neonatal sepsis, history of blood transfusion, and neonatal asphyxia were also significantly associated with an increased risk of NEC (Table 5). The Hosmer-Lemeshow test indicated no significant discrepancy between the expected and observed frequencies derived from the predicted probabilities (*χ*^2^ = 13.152, *P* = 0.071 > 0.05), confirming good model fit. A risk prediction model was constructed using the independent factors identified via multivariable logistic regression. Receiver operating characteristic (ROC) curve analysis demonstrated an area under the curve (AUC) of 0.937 (95% CI: 0.915–0.960) (Figure 2).

**Table 5:** Multivariate regression analysis of risk factors for NEC in neonates.

Variables	*β*	SE	Wald c^2^	*P*	OR	95%CI
Sepsis	0.925	0.295	9.917	0.002	2.522	1.403–4.538
Combined antibiotic use	0.683	0.212	10.28	0.001	1.976	1.305–2.972
Blood transfusion	0.781	0.297	7.015	0.008	2.183	1.245–3.809
Neonatal asphyxia	0.642	0.285	5.128	0.024	1.887	1.085–3.274
GDM	0.608	0.258	5.632	0.018	1.824	1.102–3.020

GA, gestational age; GDM, gestational diabetes mellitus.

## 4 Discussion

Based on a retrospective cohort of 344 preterm infants (172 cases in the NEC group and 172 cases in the control group), this study systematically identified potential risk factors for NEC using univariate and multivariate logistic regression analyses. A risk prediction model with excellent discriminatory power was developed, with an area under the receiver operating characteristic curve (AUC) of 0.937. These findings provide novel evidence to inform the clinical prevention and targeted management of NEC in preterm infants.

Our study suggests that neonatal sepsis is a significant independent risk factor for NEC, with an OR of 2.522 (95% CI: 1.403–4.538) for the occurrence of NEC. This finding is consistent with the observations reported by Gagliardi et al. (13). When comparing domestic and international studies investigating the association between sepsis and NEC, we observed that the odds ratio (OR) for sepsis as a risk factor for NEC reported in international literature is predominantly greater than 10 (14), while the OR values reported in China were mostly 3 to 5 (15), and the OR values of our study was 2.52. The marked discrepancy observed regarding sepsis highlights the need for caution when interpreting findings from epidemiological studies and medical statistical analyses. Sepsis can induce the massive proliferation of diverse bacteria in the bloodstream, leading to the release of substantial quantities of toxins that translocate to the intestine via the circulatory system, impair systemic negative feedback mechanisms, and ultimately result in intestinal mucosal necrosis (16). From a pathophysiological perspective, bacterial endotoxins [e.g., lipopolysaccharide (LPS)] during sepsis can activate the Toll-like receptor 4 (TLR4) signaling pathway, trigger excessive intestinal mucosal inflammation, disrupt the tight junctions of intestinal epithelial cells, and ultimately culminate in intestinal mucosal necrosis (17). Accumulating evidence indicates that TLR4 contributes to NEC pathogenesis through multiple mechanisms, including apoptosis, necroptosis, and autophagy, all of which induce intestinal barrier impairment (18, 19). Building on these mechanistic insights, intestinal-targeted strategies targeting TLR4 may represent a novel preventive approach for NEC in preterm infants (20). For instance, it has been documented that fetal intestinal lumen exposure to amniotic fluid inhibits TLR4 activation, thereby reducing the risk of NEC (21). A recent study (22) suggested a NEC incidence rate of 34%–57% among neonates with sepsis, underscoring that neonates with sepsis are at significantly increased risk of developing NEC due to impaired intestinal barrier function. Therefore, we propose enhancing systematic intestinal function monitoring (e.g., assessment of abdominal distension, stool characteristics, and feeding tolerance) in septic neonates and initiating intestinal protective interventions at the earliest feasible opportunity.

Whether blood transfusion in preterm infants increases the incidence of NEC remains controversial. The results of our study indicate that blood transfusion is an independent risk factor for NEC in preterm infants (OR = 2.18, 95% CI: 1.245–3.809). In contrast, a recent meta-analysis showed that blood transfusion does not increase the risk of NEC (23). Blood transfusion, particularly large amounts of red blood cell, can impair the regulatory function of mesenteric blood vessels, leading to increased RBC adhesion and aggregation, thrombosis formation, and subsequent intestinal hypoperfusion (24). However, a meta-analysis conducted by Rai et al. (25) demonstrated that RBC transfusion within 48 h exerts a protective effect in infants with NEC. Patel et al. (26) reported that severe anemia, rather than RBC transfusion, is associated with an elevated risk of NEC, and suggested that preventing anemia may be more beneficial than reducing RBC transfusion. Therefore, the effects of common neonatal physiological conditions (i.e., anemia) and clinical interventions (i.e., RBC transfusion) on the susceptibility of preterm infants to NEC warrant further investigation. Plausible mechanisms underlying these potential associations may involve the impacts of anemia and RBC transfusion on intestinal barrier integrity, systemic inflammation, and intestinal tissue hypoxia—processes that could be mediated by alterations in endogenous vasoactive mediators, upregulation of TLR4, and activation of pro-inflammatory macrophages.

In the present study, neonatal asphyxia was identified as a risk factor for NEC in preterm infants (OR = 1.88, 95%CI: 1.085–3.274). Preterm infants are highly susceptible to asphyxia after birth due to immature development of the respiratory and nervous systems, which impairs their ability to perform effective gas exchange independently. Neonatal asphyxia can trigger the body's defensive reflexes, leading to redistribution of systemic blood flow to prioritize oxygen supply to vital organs (e.g., the heart, brain, and kidneys). This process induces intense constriction of mesenteric blood vessels, resulting in ischemia and hypoxia of intestinal epithelial cells, and even cellular degeneration and necrosis, ultimately contributing to NEC development (27).

In the present study, gestational diabetes mellitus (GDM) was also identified as an independent risk factor for NEC in preterm infants (OR = 1.824, 95% CI: 1.102–3.020), which is consistent with the findings of a meta-analysis by Lu et al. (28). This may be attributed to the abnormal intrauterine environment caused by maternal hyperglycemia. Sustained maternal hyperglycemia can interfere with fetal intestinal hemodynamics and lead to early oxidative stress damage in intestinal tissues. This “intrauterine imprinting” may reduce the preterm infant's tolerance to enteral nutrition or bacterial colonization after birth, making them more susceptible to intestinal necrosis under stress. Maternal GDM is characterized by a hyperglycemic state. Since the fetus obtains nutrients directly from the mother, sustained exposure to maternal hyperglycemia may alter fetal intestinal blood flow dynamics and predispose the preterm infant to NEC. Specifically, sustained maternal hyperglycemia stimulates the hyperplasia of fetal pancreatic islet cells, leading to excessive fetal insulin secretion. This hyperinsulinemia can alter the sensitivity of mesenteric blood vessels to vasoconstrictors, resulting in reduced intestinal perfusion and localized tissue hypoxia. Furthermore, this abnormal intrauterine environment induces early oxidative stress, which may diminish the preterm infant's tolerance to postnatal bacterial colonization and enteral nutrition, thereby increasing susceptibility to NEC (29). This finding underscores the importance of enhancing prenatal assessment of intestinal development in fetuses of mothers with GDM.

The impact of antibiotics on the development of NEC remains controversial in current research. For instance, a recent multicenter birth cohort study of very low birth weight infants (30) found that infants who did not receive antibiotic treatment after birth had a higher incidence of NEC. Berkhout et al. (22) demonstrated that among preterm infants with a GA ≤ 30 weeks, antibiotic administration within 24 h after birth reduced the risk of NEC. However, this protective effect appears to be time-dependent: with the unnecessary prolongation of antibiotic treatment duration, the risk of NEC gradually emerges. Specifically, for preterm infants without confirmed infection (31), prolonging initial empirical antibiotic therapy may increase the risk of NEC or death, and has been independently associated with NEC development.Our study identified combined antibiotic use as an independent risk factor for NEC (OR = 1.976). This finding suggests that while antibiotics are essential for managing neonatal infections, their adverse impact on the intestinal environment cannot be overlooked. Compared to monotherapy, combination therapy typically possesses a broader antibacterial spectrum, exerting a “carpet-bombing” effect on the immature gut microbiota of preterm infants. Specifically, the concurrent administration of broad-spectrum antibiotics significantly reduces species richness and microbial diversity within the neonatal gut. This “colonization pressure” leads to a profound depletion of protective commensal bacteria, such as Bifidobacterium and Lactobacillus, while facilitating the overproliferation of opportunistic pathogens. Evidence suggests that the loss of microbial diversity compromises the integrity of the intestinal epithelial barrier and triggers an intense inflammatory cascade via the activation of the Toll-like receptor 4 (TLR4) signaling pathway, ultimately culminating in mucosal injury and necrosis (32). Collectively, these findings underscore that antibacterial agents act as a double-edged sword in preterm infants. Given their immature immune function, short-term rational antibiotic use can reduce infection-related mortality; however, inappropriate use (e.g., prolonged courses, unnecessary broad-spectrum therapy) imposes unnecessary burdens on this vulnerable population and increases the risk of in-hospital complications (33). Therefore, clinicians should strengthen antibiotic stewardship in preterm infants, strictly adhering to evidence-based principles for the timing, regimens, and durations of antibiotic administration. For preterm infants with suspected infection, short-term empirical therapy with narrow-spectrum antibacterial agents is recommended. For those with confirmed infection, clinical decisions should be tailored to individual patient conditions to avoid unnecessary prolongation of broad-spectrum antibiotic exposure.

The limitations of this study are as follows: (1) Its retrospective design may introduce information bias.Due to the retrospective design, we lacked granular data on the specific types of human milk (e.g., Mother's Own Milk vs. Pasteurized Donor Human Milk) and the precise dose-response relationship (e.g., the proportion of human milk in the daily diet) prior to NEC onset. This may have limited our ability to fully evaluate the protective potential of different feeding strategies. (2) As a single-center study, the sample size is relatively limited, and some significant between-group differences may have been overlooked. Furthermore, our variable selection for the multivariable model was primarily based on univariate statistical significance. While this is a standard clinical research practice, it may exclude potential confounders that are clinically relevant but did not reach the significance threshold in this specific cohort. Future studies using larger datasets and Directed Acyclic Graphs (DAGs) are needed to further refine the causal architecture of these risk factors. (3) No stratified analysis was performed based on the severity of NEC, and the identification of preterm infants with severe NEC was not explored. (4) The current risk prediction model (AUC = 0.937) was developed using the total study cohort and has not yet undergone internal cross-validation or external validation with an independent dataset. Its generalizability should be interpreted with caution until further multi-center validation is performed.

## 5 Conclusions

In conclusion, this study identifies neonatal sepsis, blood product transfusion, combined antibiotic therapy, neonatal asphyxia, and maternal GDM as independent risk factors for NEC. By eliminating the confounding effects of gestational age and birth weight through a matched design, our findings underscore the critical role of perinatal clinical interventions and maternal health in NEC pathogenesis. The developed risk prediction model (AUC = 0.937) serves as a robust tool for early identification of high-risk preterm infants. Clinicians should prioritize antibiotic stewardship and rigorous glucose management in GDM mothers to mitigate NEC risk. Future multi-institutional studies are warranted to validate the model's clinical utility across diverse neonatal populations.

## Data Availability

The original contributions presented in the study are included in the article/Supplementary Material, further inquiries can be directed to the corresponding author.
